# Measuring information flow in cellular networks by the systems biology method through microarray data

**DOI:** 10.3389/fpls.2015.00390

**Published:** 2015-06-02

**Authors:** Bor-Sen Chen, Cheng-Wei Li

**Affiliations:** Laboratory of Control and Systems Biology, Department of Electrical Engineering, National Tsing Hua UniversityHsinchu, Taiwan

**Keywords:** system theory, information flow, signal transductivity, information transductivity, microarray data, signal transduction pathway, gene regulatory network

## Abstract

In general, it is very difficult to measure the information flow in a cellular network directly. In this study, based on an information flow model and microarray data, we measured the information flow in cellular networks indirectly by using a systems biology method. First, we used a recursive least square parameter estimation algorithm to identify the system parameters of coupling signal transduction pathways and the cellular gene regulatory network (GRN). Then, based on the identified parameters and systems theory, we estimated the signal transductivities of the coupling signal transduction pathways from the extracellular signals to each downstream protein and the information transductivities of the GRN between transcription factors in response to environmental events. According to the proposed method, the information flow, which is characterized by signal transductivity in coupling signaling pathways and information transductivity in the GRN, can be estimated by microarray temporal data or microarray sample data. It can also be estimated by other high-throughput data such as next-generation sequencing or proteomic data. Finally, the information flows of the signal transduction pathways and the GRN in leukemia cancer cells and non-leukemia normal cells were also measured to analyze the systematic dysfunction in this cancer from microarray sample data. The results show that the signal transductivities of signal transduction pathways change substantially from normal cells to leukemia cancer cells.

## Introduction

No cell lives in isolation. Even eukaryotic microorganisms such as yeast, slime molds, and protozoans can secrete molecules called pheromones to coordinate the aggregation of free-living cells for sexual mating or differentiation under certain environmental conditions (Lipke and Kurjan, 1992; Figueiras et al., 2009; O'day and Keszei, 2012). The most important among these molecules are extracellular signaling molecules that function within plants and animals to control metabolic processes within cells, the growth and differentiation of tissues, the synthesis and secretion of proteins, and the composition of the intracellular and extracellular fluids. During intercellular communication or cellular stress responses, the cell senses extracellular signals. Different external changes or events may stimulate signaling. Typical signals are hormones, pheromones, heat, cold, light, osmotic pressure, and the appearance or changes in the concentration of substances such as glucose, potassium ions, calcium ions, or cyclic adenosine monophosphate (cAMP) (Dibner et al., 2010; Dodd et al., 2010; Kim and Choi, 2010; Kim et al., 2010; Leung and Sharp, 2010; Mosenden and Tasken, 2011). In the flow of the signal transduction pathway, the extracellular signals are perceived by a transmembrane receptor. The receptor changes its own state from inactive to active and then triggers subsequent cellular processes. The active receptor stimulates an internal signaling cascade. This cascade frequently includes a series of changes in protein phosphorylation states. The changes affect downstream proteins across the nuclear membrane. Eventually, a transcription factor (TF) is activated or deactivated, which changes its binding activity to target genes that encode the corresponding proteins in response to extracellular signals or stresses. Therefore, signal transduction pathways can also be viewed as information-processing and transferring systems to control the gene activities of cells in response to stimuli (Tay et al., 2010).

With regards to information-processing and transferring systems, many studies have investigated the properties of signal transduction pathways, such as amplification(Little et al., 2011), specification (Corada et al., 2010), adaptive ultrasensitivity (Srividhya et al., 2011), oscillation (Waters et al., 2014), and synchronization (Liu et al., 2014). However, owing to the complex nature of dynamic networks, knowledge of their components and interactions is often not sufficient to interpret their system behavior. Therefore, it remains challenging to analyze the information flow in signal transduction pathways efficiently.

Information transmission ability was first expressed as a mathematical formula in Koshland et al. (1982). This report focused on the sensitivity amplification of signals, which is defined as the ratio of percent change in output response to percent change in input signals, i.e., the relative change in transduction system output with respect to a specific input. Signal amplification is also defined as the signal gain of the signal transduction pathways (Chen and Lin, 2012; Chen and Wu, 2012). Information flow is necessarily interpreted on a case-by-case basis, i.e., the signal transductivity measured in a signal transduction pathway is affected not only by the structure of the signal transduction system but also by the input to the signal transduction system.

In this study, the information flows of both coupling signal transduction pathways and the downstream gene regulatory network (GRN) were estimated by system identification techniques and a systems biology method using microarray data. First, the dynamic information flow model of coupling transduction pathways was identified by microarray temporal data. Then, based on a system theory about the discrete-time state-space model, the signal transductivity was estimated for each of the coupling transduction pathways from receptors at the membrane to TFs in the nucleus from a system gain perspective. When the microarray data were not time-profile data, but rather represented different samples at a single time point, the information flow for the coupling signal transduction pathway was also estimated based on a linear regression model and the recursive least square parameter estimation method.

In general, it is challenging to calculate the information transductivity from one gene to another in a GRN from graph theory, especially with a large directed graph (digraph). In this study, instead of using the digraph method, the regulatory dynamic model was rearranged as a dynamic state-space system with a single gene as an input and another as an output. Then, based on the state-space dynamic system theory and the recursive parameter estimation method, we estimated the information transductivity of a GRN from one gene to another from the microarray temporal data. Similarly, when the microarray data represented a single time point but different samples, we estimated the information transductivity between genes in the GRN based on the steady-state model.

Finally, we used microarray sample data to estimate the information flow in cancer-related signal transduction pathways and a GRN. We compared the information flows of signal transduction pathways between leukemia cancer cells and non-leukemia normal cells. Based on the information flow analysis, we traced back the main cause of systematic dysfunction of the related proteins in the signal transduction pathways. Furthermore, we also identified the systematic dysfunction of information flow between genes related to leukemogenesis. The methods proposed here are very useful for estimating signal transductivity or information transductivity in cellular systems using microarray temporal or sample data. Since the information flow model can also be identified using high-throughput data such as next-generation sequencing (NGS) or proteomic data, the proposed method can also be used to estimate signal transductivity and information transductivity from NGS or proteomic data efficiently in future.

System identification technologies for discrete-time systems and linear matrix inequalities are standard methods, which, when combined with system gain theories and microarray data to estimate the information flow in signaling pathways in cellular systems, provide a new method for measuring information flow in cellular networks using microarray data. To the best of our knowledge, this is the first study to quantify the information flow between large coupling signal transduction pathways and a complex GRN using corresponding microarray data.

The proposed method has the following limitations. (1) We use a linear model to approximately measure the information flow. (2) Proteomic data that comprise differential expressions are not discussed in this study. Although the information flow can be measured using a non-linear model, the parameter estimation of the networks will be more difficult due to the non-linearity of the model, and more samples of microarray data are required for the identification of more parameters in a non-linear model. Furthermore, the information flow also becomes more difficult to measure in coupling signaling pathways and a GRN. Additionally, the results measured by the proposed methods will be biased by microarray data noise. In other words, the variance of parameter estimation error, i.e., the bias of the proposed least square parameter estimation, is proportional to the variance of microarray data noise (Johansson, 1993).

## The information flow in signal transduction pathways

### Estimation of signal transductivity by microarray temporal data

**Figure 1:**
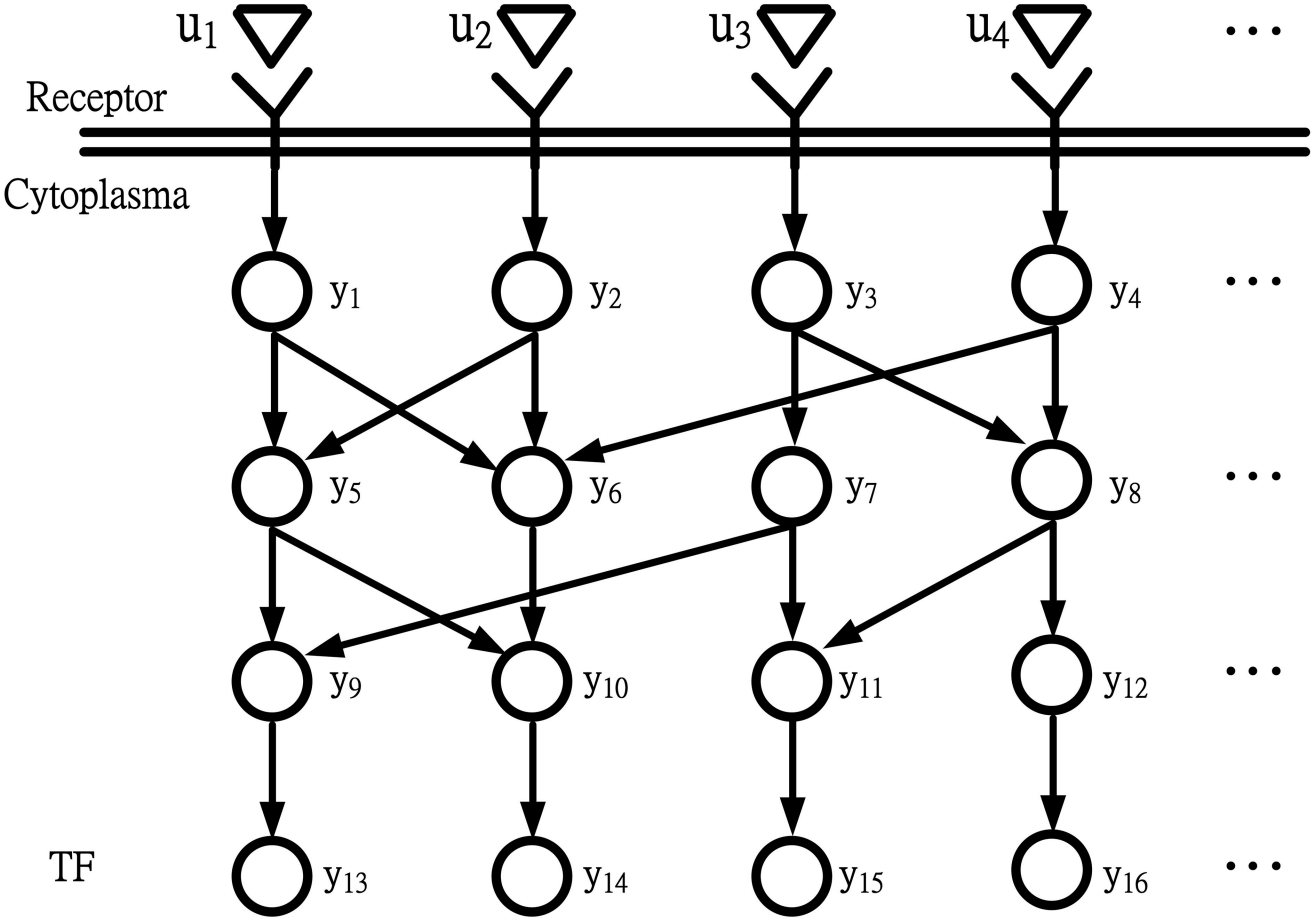
**Information flow chart depicting coupling signal transduction pathways that control gene activities**. *u*_1_, *u*_2_, *u*_3_, and *u*_4_ are extracellular signals; *y*_13_, *y*_14_, *y*_15_, and *y*_16_ are expression levels of TFs.

For the coupling signal transduction pathways in Figure 1 throughout intercellular communication or cellular stress response, the receptors in the cell membrane sensed extracellular signals. *y_i_*(*t*) denotes the expression level of the *i*th protein in the coupling signal transduction pathways. They are commuted to intracellular signals and sequences of reactions. *u_i_*(*t*) denotes extracellular signals. Different external changes or events outside the cell may stimulate signaling. Typical extracellular signals are hormones, pheromones, heat, cold, light, osmotic pressure, and appearance or concentration change of substance such as glucose, potassium ion, calcium ion, or cAMP (Klipp, 2005; Lin et al., 2007; Dibner et al., 2010; Dodd et al., 2010; Kim and Choi, 2010; Kim et al., 2010; Leung and Sharp, 2010; Li and Chen, 2010; Mosenden and Tasken, 2011). The extracellular signals *u_i_*(*t*) for *i* = 1,…,*l* are perceived by a transmembrane receptor, as depicted in Figure 1. The receptor changes its own state from susceptible to active and then triggers subsequent processes within the cell. The active receptor stimulates an internal signaling cascade. This cascade frequently includes a series of changes in protein phosphorylation states. The changes affect downstream proteins across the nuclear membrane. Eventually, the TF is activated or deactivated to change its binding activity to target genes. In the information flow chart of simple coupling transduction pathways in Figure 1, *y*_13_(*t*), *y*_14_(*t*), *y*_15_(*t*), and *y*_16_(*t*) represent the expression levels of terminal TFs in the simple coupling signal transduction pathways.

For the purpose of system identification for the information flow of coupling signal transduction pathways in Figure 1, a simple regression model for the expression level of the *i*th protein at time *t* + 1 can be described as follows:

(1) $$\begin{array}{l} y_{i}(t\;+\;1)\;=\;c_{i,\;1}y_{1}(t)\;+\;\cdots \;+\;c_{i,\;i-1}y_{i-1}(t)\;+\;c_{i,\;i}y_{i}(t)\\ \;+\;c_{i,i+1,}y_{i+1}(t)+\cdots +c_{i,M}y_{M}(t)+h_{i}\\ \;+\sum_{j=1}^{l}b_{i,\;j}u_{j}(t)\;+\;w_{i}(t),\text{ for }i\text{= 1},\;\cdots ,\;M \end{array}$$

where *y_i_*(*t*) indicates the expression level of the *i*th protein at time *t*; *c_i, j_* denotes the interaction ability between protein *i* and protein *j*; *h_i_* denotes the basal level of the *i*th protein; and *b_ij_* denotes the binding ability of extracellular signal *j* to the *i*th protein. In general, extracellular signals always bind to the receptor proteins on the membrane.

Let us denote the state vector and system matrix of coupling signal transduction pathways of Figure 1 in Equation (1) as follows:

$$\begin{array}{l} y(t)\;=\;\left[\begin{array}{c} y_{1}(t)\\ \vdots \\ y_{M}(t) \end{array}\right],\;C\;=\;\left[\begin{array}{ccc} c_{1,\;1} & \cdots & c_{1,\;M}\\ \vdots & \ddots & \vdots \\ c_{M,\;1} & \cdots & c_{M,\;M} \end{array}\right],\\ H\;=\;\left[\begin{array}{c} h_{1}\\ \vdots \\ h_{M} \end{array}\right],\;B\;=\;\left[\begin{array}{ccc} b_{1,\;1} & \cdots & b_{1,\;l}\\ \vdots & \ddots & \vdots \\ b_{M,\;1} & \cdots & b_{M,\;l} \end{array}\right],\\ u(t)\;=\;\left[\begin{array}{c} u_{1}(t)\\ \vdots \\ u_{l}(t) \end{array}\right],\;w(t)\;=\;\left[\begin{array}{c} w_{1}(t)\\ \vdots \\ w_{M}(t) \end{array}\right] \end{array}$$

Then, the network of coupling signal transduction pathways in Figure 1 is represented by:

(2) y(t + 1) = Cy(t) + H + Bu(t) + w(t)

To exploit the effect of extracellular signals *u*(*t*) on the coupling signal transduction pathways, the effect of basal level *H* should be extracted from the dynamic network in Equation (2). Without consideration of extracellular signal *u*(*t*), the network of coupling signal transduction pathways in Equation (2) can be represented by:

(3) $$\hat{y}(t+1)\;=\;C\hat{y}(t)+H+w(t)$$

which is only the effect of basal level *H* and noise *w*(*t*).

Let us denote $\tilde{y}$(*t*) = *y*(*t*)−$\hat{y}$(*t*) and subtract Equation (3) from Equation (2). We then get

(4) $$\tilde{y}(t+1)\;=\;C\tilde{y}(t)+Bu(t)$$

where $\tilde{y}$(*t*) denotes the information flow in the coupling signal transduction pathways due to extracellular signals *u*(*t*) in Figure 1.

In this case, the initial condition is at zero, $\tilde{y}$(0) = 0. The solution of the recursive dynamic equation in (4) is given by the following information flow equation (Ogata, 1987):

(5) $$\tilde{y}(t+1)\;=\;\sum_{j=0}^{t}C^{t-j}Bu(j)$$

Obviously, if the system matrices *C* and *B* of the coupling signal transduction pathways can be identified from the microarray data, the information flow in the input/output response equation in (5) can be calculated.

Let us denote the signal transduction level ρ of the coupling signal transduction pathways from the extracellular signals *u*(*t*) to $\tilde{y}$(*t*) in Equation (4):

(6) $$\frac{\sum_{t=0}^{t_{p}}{\tilde{y}}^{T}(t)\tilde{y}(t)}{\sum_{t=0}^{t_{p}}u^{T}(t)u(t)}\;\leq \;\rho ,\;\forall u(t)\in l_{2}[0,\;t_{p}]$$

where *t_p_* denotes the present time; *l*_2_[0, *t_p_*] denotes the set of all possible extracellular signals with bounded energy in [0, *t_p_*]; and ρ denotes the upper bound of the signal transductivity of the coupling signal transduction pathways.

**Proposition 1:**

The coupling signal transduction pathways in Equation (4) have a signal transduction level ρ in Equation (6) if the following linear matrix inequality (LMI) holds for some positive definite matrix *P* = *P^T^* > 0

(7) $$\left[\begin{array}{cc} C^{T}PC-P+I & C^{T}PB\\ B^{T}PC & B^{T}PB-\rho I \end{array}\right]\leq 0$$

Proof: see Appendix A.

Since ρ is the upper bound of signal transductivity ρ_0_ of the coupling signal transduction pathways from extracellular signals *u*(*t*) to $\tilde{y}$(*t*)in Equation (4), the signal transductivity of the signal transduction network in Figure 1 can be obtained by solving the following LMI-constrained optimization problem:

(8) $$\begin{array}{c} \rho_{0}\;=\;\underset{P>0}{\min}\rho \\ \text{subject to LMI in }(\text{7}) \end{array}$$

The constraint optimization problem in Equation (8) can be easily solved by decreasing the upper bound ρ in Equation (7) until no *P* > 0 exists in Equation (7) by using the MATLAB LMI toolbox.

**Remark 1**: The signal transductivity ρ_0_ in Equation (8) is equivalent to the system gain from *u*(*t*) to $\tilde{y}$(*t* + 1) in Equation (4) (Boyd, 1994; Chiu and Chen, 2011):

(9) $$\rho_{0}=\underset{u(t)\in l_{2}[0,\;t_{p}]}{\sup}\frac{{\left\|\tilde{y}(t+1)\right\|}_{2}}{{\left\|u(t)\right\|}_{2}}$$

from a system theory perspective.

If we want to know the information flow from *u*(*t*) to any protein, then the coupling signal transduction dynamic equation in (4) should be represented by

(10) $$\begin{array}{l} \tilde{y}(t+1)\;=\;C\tilde{y}(t)+Bu(t)\\ {\tilde{y}}_{i}(t)\;=\;[\underset{1\sim (i-1)}{\underset{︸}{0}}1\underset{(i+1)\sim M}{\underset{︸}{0}}]\tilde{y}(t)\;=\;D_{i}\tilde{y}(t) \end{array}$$

where $\tilde{y}$*_i_*(*t*) denotes the expression of the *i*th protein and *D_i_* is a row vector with all zeros except 1 at the *i*th element.

**Remark 2**: (i) The information flow from *u*(*t*) to $\tilde{y}$*_i_*(*t*) in the signal transduction dynamic equation in (10) is given by ${\tilde{y}}_{i}(t+1)\;=\;\sum_{j=0}^{t}D_{i}C^{t-j}Bu(j),\text{ for }i=1,\;\ldots ,\;M.$

In this situation, the signal transductivity ρ*^i^*_0_ from *u*(*t*) to the *i*th protein in the coupling signal transductivity pathways is solved by the following LMI-constrained optimization problem:

(11) $$\begin{array}{c} \rho_{o}^{i}=\begin{array}{cc} \underset{P>0}{\min} & \rho \end{array}\\ \text{subject to }\left[\begin{array}{cc} C^{T}PC-P+D_{i}^{T}D_{i} & C^{T}PB\\ B^{T}PC & B^{T}PB-\rho I \end{array}\right]\;\leq \;0 \end{array}$$

(ii) Further, if we only want to discuss the signal transductivity from the *l* extracellular signals to the *i*th protein, and we let *B_l_* = [*b*_1_*_l_* … *b_Ml_*]*^T^*, the binding vector of the *l*th extracellular signal to the *M* proteins of the coupling signal transduction pathway, it can be calculated by solving the following LMI-constrained optimization problem:

(12) $$\begin{array}{c} \rho_{o}^{i,l}=\begin{array}{cc} \underset{P>0}{\min} & \rho \end{array}\\ \text{subject to }\left[\begin{array}{cc} C^{T}PC-P+D_{i}^{T}D_{i} & C^{T}PB_{l}\\ B_{l}^{T}PC & B_{l}^{T}PB_{l}-\rho I \end{array}\right]\leq 0 \end{array}$$

(iii) If the NGS data and proteomic data are available, epigenetic regulations, such as DNA methylation and histone modification, can be also involved in our model. The regulations are considered as the extra inhibition term −*B_m_m*(*t*) in Equation (2). *m*(*t*) denotes the expressions of DNA methylation or histone. In this case, Equation (2) is modified as:

$$y(t+1)\;=\;Cy(t)+H+Bu(t)-B_{m}m(t)\;+\;w(t)$$

We then obtain

$$y(t+1)\;=\;\sum_{j=0}^{t}C^{t-j}\left(Bu(j)-B_{m}m(j)+H+w(j)\right)$$

It does not influence signal transductivity ρ_0_ from *u*(*t*) to *y*(*t*) in Equation (9) but will influence output signal *y*(*t*).

(iv) The above LMI-constrained optimization problem can be easily solved with the help of LMI solver in the MATLAB LMI toolbox by decreasing ρ until no *P* > 0 exists in the LMI. The LMI solver works essentially in four steps, initial guess, elimination of equality constraints, elimination of variables, and optimization. The pipeline description of the procedure to solve the LMI-constrained optimization problem in Equation (12) is available (Elghaoui et al., 1995).

Based on the above analyses, if the system matrices *C, H*, and *B* can be identified from the microarray temporal data or proteomic temporal data, the signal flow in Equation (5) and the signal transductivity ρ_0_ in Equation (8), (11), or (12) can be easily estimated by solving the corresponding LMI-constrained optimization problem. Therefore, as follows, we will focus on how to estimate these system matrices of coupling signal transduction pathways in Equation (2) from microarray data. In general, we do not identify *C, H*, and *B* from Equation (2) directly owing to its complex computation with much more round off error in the parameter identification process. We want to estimate these parameters protein-by-protein from Equation (1). From Equation (1), they can be represented by the following regression form:

(13) $$\begin{array}{l} y_{i}(t+1)\;=\;[y_{1}(t)\;\cdots \;y_{M}(t)\;u_{1}(t)\;\cdots \;u_{l}(t)1]\text{​}\left[\begin{array}{c} c_{i,1}\\ \vdots \\ c_{i,M}\\ b_{i,1}\\ \vdots \\ b_{i,l}\\ h_{i} \end{array}\right]\text{​ }+\;w_{i}(t)\\ ≜\phi_{i}(t)\theta_{i}+w_{i}(t),\text{ for }i\;=\;1,\cdots ,M \end{array}$$

By the recursive least square parameter estimation algorithm (Johansson, 1993),

(14) $$\begin{array}{cccc} \theta_{i}(t+1) & = & \theta_{i}(t)+P_{i}(t)\phi_{i}(t)(y_{i}(t)-\phi_{i}^{T}(t)\theta_{i}(t-1)) & \\ P_{i}(t) & = & P_{i}(t-1)-\frac{P_{i}(t-1)\phi_{i}(t)\phi_{i}^{T}(t)P_{i}(t-1)}{1+\phi_{i}^{T}(t)P_{i}(t-1)\phi_{i}(t)}, & \\ \text{for }i & = & \begin{array}{l} 1,\cdots ,M\\ \theta_{i}(0)\text{ and }P_{i}(0)\text{ are given} \end{array} & \end{array}$$

This recursive least square parameter estimation can use microarray temporal data to update parameters protein-by-protein. Therefore, it can be used for real-time parameter estimation. If the number of time-profile data *y_i_*(*t*) is small, we can repeat several rounds of the recursive parameter estimation algorithm in Equation (14) with the previous result as initial parameter estimate θ_i_(0) and initial *P_i_*(0) to achieve the optimal parameter estimate. After parameter estimate θ*_i_* from the least square parameter estimation algorithm in Equation (14) for all proteins in the coupling signal transduction pathways, i.e., *i* = 1,…,*M*, we can estimate the system matrices *C, H*, and *B* of both coupling signal transduction pathways in Equation (2) from microarray temporal data. By substituting these system parameters into the constrained optimization problem in Equation (8), (11), or (12), we can estimate different kinds of signal transductivities from the extracellular signals to proteins in the coupling signal transduction pathways through microarray temporal data.

### Estimation of signal transductivity by microarray sample data

If the microarray data for measuring signal transductivity is of one time-point microarray from different samples, the regression model for the protein expression level of the *i*th protein in the coupling signal transduction pathways in Figure 1 cannot be represented by the discrete-time dynamic equation in (2) but can be represented by the following linear static regression form:

(15) y(k) = Cy(k)+H+Bu(k)+w(k), k = 1, …, K

where *y*(*k*) = [*y*_1_(*k*) … *y_M_*(*k*)]*^T^*; *u*(*k*) = [*u*_1_(*k*) … *u_l_*(*k*)]^*T*^; *w*(*k*) = [*w*_1_(*k*) … *w_M_*(*k*)]*^T^*; *C, H*, and *B* are defined as in Equation (2); and *k* = 1,…,*K* denote the samples of microarray data.

In this situation,

(16) $$y(k)\;=\;{\left(I-C\right)}^{-1}Bu(k)+{\left(I-C\right)}^{-1}H+{\left(I-C\right)}^{-1}w(k)$$

From Equation (16), it is seen that the transduction function *T* from *u* to *y* is

(17) $$T={\left(I-C\right)}^{-1}B$$

Then, the signal transductivity is obtained as Boyd (1994):

(18) $$\rho_{0}=\sup\frac{{\left\|y(k)\right\|}_{2}}{{\left\|u(k)\right\|}_{2}}\;=\;{\left\|T\right\|}_{2}\;=\;{\left\|{\left(I-C\right)}^{-1}B\right\|}_{2}\;=\;\sigma_{\max}({\left(I-C\right)}^{-1}B)$$

where σ_max_(·) denotes the maximum singular value.

Therefore, if we want to estimate the signal transduction function *T* in Equation (17) or the signal transductivity in Equation (18), we need to estimate the system matrices *C* and *B* in Equation (15) from the microarray sample data. From the linear regression model in Equation (15), we get:

(19) $$\begin{array}{l} y_{i}(k)\;=\;[y_{1}(k)\;\cdots \;y_{M}(k)\;u_{1}(k)\;\cdots \;u_{l}(k)1]\left[\begin{array}{c} c_{i,\;1}\\ \vdots \\ c_{i,\;M}\\ b_{i,\;1}\\ \vdots \\ b_{i,\;l}\\ h_{i} \end{array}\right]\;+\;w_{i}(k)\\ ≜\phi_{i}(k)\theta_{i}+w_{i}(k),\text{ for }k\;=\;1,\;\cdots ,\;K \end{array}$$

The recursive least square parameter identification for θ*_i_* of the *i*th protein in Equation (19) with *K* sample microarray is given by Johansson (1993):

(20) $$\begin{array}{l} \theta_{i}(k)\;=\;\theta_{i}(k-1)+P_{i}(k)\phi_{i}(k)\varepsilon_{i}(k)\\ \varepsilon_{i}(k)\;=\;(y_{i}(k)-\phi_{i}^{T}(k)\theta_{i}(k-1)),\;\theta_{i}(0)\text{ and }P_{i}(0)\text{ are given}\\ P_{i}(k)\;=\;P_{i}(k-1)-\frac{P_{i}(k-1)\phi_{i}(k)\phi_{i}^{T}(k)P_{i}(k-1)}{1+\phi_{i}^{T}(k)P_{i}(k-1)\phi_{i}(k)},\\ \text{ for }i\;=\;1,\;\cdots ,\;M,\text{ and }k\;=\;1,\;\cdots ,\;K \end{array}$$

If the sample number of microarray data is small, we can repeat several rounds of the recursive parameter estimation algorithm in Equation (20) with previous results as initial conditions θ*_i_*(0) and *P_i_*(0) to achieve the optimal parameter estimate. After the parameters θ*_i_* for *i* = 1,…,*M* are estimated by the recursive least square estimation algorithm in Equation (20) through microarray data with *K* samples, we can estimate *C, H*, and *B* in Equation (15). Then, the signal transduction function *T* in Equation (17) or the signal transductivity ρ_0_ in Equation (18) can be calculated through microarray sample data. If we only want to estimate the signal transduction from all extracellular signals *u*(*k*) to the *i*th protein (or TF) *y_i_*(*k*), then *T* in Equation (17) should be replaced by:

(21) $$\frac{y_{i}(k)}{u(k)}\;=\;T_{i}\;=\;D_{i}{\left(I-C\right)}^{-1}B$$

where *D_i_* is defined in Equation (10).

The signal transductivity from all extracellular signals *u*(*k*) to the *i*th protein is given by:

(22) $$\rho_{0}^{i}\;=\;\sigma_{\max}(D_{i}{\left(I-C\right)}^{-1}B)\;=\;{\left\|D_{i}{\left(I-C\right)}^{-1}B\right\|}_{2}$$

where || · ||_2_ denotes 2-norm.

**Remark 3**: If we only want to estimate the information flow from the *l*th extracellular signal *u_l_*(*k*) to the *i*th protein (TF) *y_i_*(*k*), then *T* in Equation (21) should be replaced by:

(23) $$\frac{y_{i}(k)}{u_{l}(k)}\;=\;T_{i,\;l}\;=\;D_{i}{\left(I-C\right)}^{-1}B_{l}$$

and the signal transductivity from *u_l_*(*k*) to the *i*th protein is given by:

$$\rho_{0}^{i,\;l}\;=\;\sigma_{\max}(D_{i}{\left(I-C\right)}^{-1}B_{l})\;=\;\left|D_{i}{\left(I-C\right)}^{-1}B_{l}\right|$$

where | · | denotes the absolute value.

## The information flow in a GRN

### Estimation of information transductivity of a GRN by microarray temporal data

**Figure 2:**
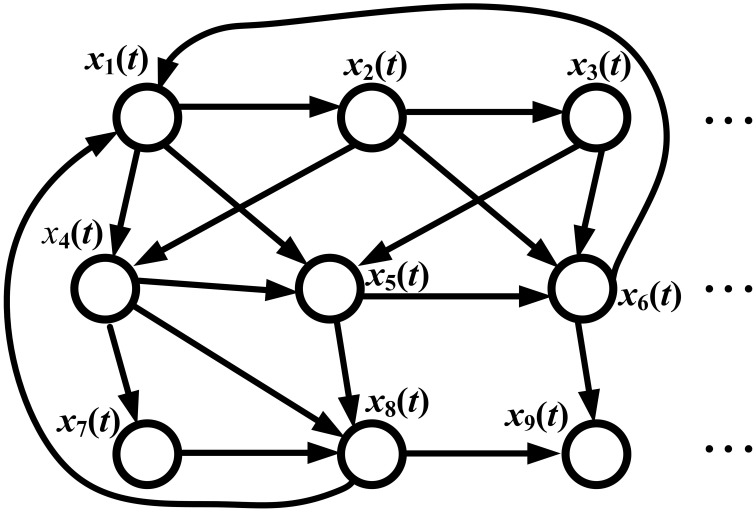
**Information flow in a GRN**. *x_i_*(*t*) denotes the gene expression of the *i*th gene. Since the gene regulation is directional, the graph of the GRN is a directed graph (digraph). The signal processing model of the GRN is given in Equation (26) by microarray temporal data; the signal flow between any two genes is described by Equation (28) and the information transductivity can be obtained by solving Equation (30). If data are available from only one temporal sample microarray, the static state-space model in Equation (32) is used, and the information flow between any two genes is given by Equation (34), with information transductivity in Equation (36).

Consider the information flow of the GRN in Figure 2. The regulatory dynamics of the *i*th gene can be represented by the following regressive equation

(24) $$x_{i}(t+1)\;=\;a_{i,\;1}x_{1}(t)+\;\cdots \;+a_{i,\;n}x_{n}(t)+v_{i}(t)$$

where *x_i_*(*t*) denotes the gene expression level of the *i*th gene; *v_i_*(*t*) denotes noise and residue; and *a_i_, _j_* denotes the regulatory ability of gene *j* on gene *i*.

**Remark 4**: (i) In general, a positive value of *a_i, j_* means the *j*th gene is an active regulator while a negative value of *a_i, j_* means the *j*th gene is an inhibitive regulator. (ii) The regulatory parameter *a_i, j_*, for *j* = 1,…, *n*, in Equation (24) can be identified by the recursive least square parameter estimation algorithm in Equation (14) through the corresponding microarray temporal data *x_i_*(*t*), for *i* = 1,…, *n* (Chen and Wang, 2006).

Therefore, the GRN in Figure 2 can be represented as follows:

(25) $$\left[\begin{array}{l} x_{1}(t+1)\\ \vdots \\ x_{i}(t+1)\\ \vdots \\ x_{n}(t+1) \end{array}\right]\;=\;\left[\begin{array}{lllll} a_{1,\;1} & \cdots & a_{1,\;j} & \cdots & a_{1,\;n}\\ \vdots & \ddots & \vdots & \ddots & \vdots \\ a_{i,\;1} & \cdots & a_{i,\;j} & \cdots & a_{i,\;n}\\ \vdots & \ddots & \vdots & \ddots & \vdots \\ a_{n,\;1} & \cdots & a_{n,\;j} & \cdots & a_{n,\;n} \end{array}\right]\;\left[\begin{array}{l} x_{1}(t)\\ \vdots \\ x_{j}(t)\\ \vdots \\ x_{n}(t) \end{array}\right]\;+\;\left[\begin{array}{l} v_{1}(t)\\ \vdots \\ v_{i}(t)\\ \vdots \\ v_{n}(t) \end{array}\right]$$

(26) X(t + 1) = AX(t) + v(t)

Suppose we want to estimate the information transductivity from gene *j* to gene *i*. In general, it is difficult to solve the information flow problem for the GRN in Figure 2 from the graph theory perspective (Kreyszig, 1993), especially for a digraph (directed graph) like Figure 2. In this study, an input/output state-space method is proposed to solve this difficult information flow problem of the digraph as follows. First, the dynamic model of the GRN in Equation (25) is represented by the following input/output dynamic state-space equation (Ogata, 1987):

(27) $$\begin{array}{l} \left[\begin{array}{l} x_{1}(t+1)\\ \vdots \\ x_{i-1}(t+1)\\ x_{i}(t+1)\\ x_{i+1}(t+1)\\ \vdots \\ x_{n}(t+1) \end{array}\right]\;=\;\left[\begin{array}{lllllll} a_{1,\;1} & \cdots & a_{1,\;i} & \cdots & a_{1,\;j} & \cdots & a_{1,\;n}\\ \vdots & \ddots & \vdots & \ddots & \vdots & \ddots & \vdots \\ a_{i-1,\;1} & \cdots & a_{i-1,\;i} & \cdots & a_{i-1,\;j} & \cdots & a_{i-1,\;n}\\ a_{i,\;1} & \cdots & a_{i,\;i} & \cdots & a_{i,\;j}-1 & \cdots & a_{i,\;n}\\ a_{i+1,\;1} & \cdots & a_{i+1,\;i} & \cdots & a_{i+1,\;j} & \cdots & a_{i+1,\;n}\\ \vdots & \ddots & \vdots & \ddots & \vdots & \ddots & \vdots \\ a_{n,\;1} & \cdots & a_{n,\;i} & \cdots & a_{n,\;j} & \cdots & a_{n,\;n} \end{array}\right]\\ \;\left[\begin{array}{l} x_{1}(t)\\ \vdots \\ x_{i}(t)\\ \vdots \\ x_{j}(t)\\ \vdots \\ x_{n}(t) \end{array}\right]\;+\;\left[\begin{array}{l} 0\\ \vdots \\ 0\\ 1\\ 0\\ \vdots \\ 0 \end{array}\right]x_{j}(t)+\left[\begin{array}{l} v_{1}(t)\\ \vdots \\ v_{i-1}(t)\\ v_{i}(t)\\ v_{i+1}(t)\\ \vdots \\ v_{n}(t) \end{array}\right]\\ \;x_{i}(t)\;=\;\left[\begin{array}{ccccccc} 0 & \cdots & 0 & 1 & 0 & \cdots & 0 \end{array}\right]\left[\begin{array}{l} x_{1}(t)\\ \vdots \\ x_{i-1}(t)\\ x_{i}(t)\\ x_{i+1}(t)\\ \vdots \\ x_{n}(t) \end{array}\right] \end{array}$$

In the input/output dynamic state-space system (Equation 27), *x_j_*(*t*) is considered an input signal and *x_i_*(*t*) an output signal. Therefore, Equation (27) is simply represented by:

(28) $$\begin{array}{c} X(t+1)\;=\;A_{j}X(t)+B_{j}x_{j}(t)+v(t)\\ x_{i}(t)\;=\;D_{i}X(t) \end{array}$$

which is similar to the signal transduction dynamic equation in (10) but with the gene expression level *x_j_*(*t*) replacing the extracellular signal *u*(*t*).

By the system theory (Ogata, 1987; Johansson, 1993), the information flow from gene *j* to gene *i* in the GRN in Figure 2 is given by

(29) $$x_{i}(t)=\sum_{k=0}^{t}D_{i}A_{j}^{t-k}B_{j}x_{j}(k)$$

Similar to solving the LMI-constrained optimization problem in Equation (12) for the information transductivity from the *l*th extracellular signal to the *j*th protein, the information transductivity γ_0_*^i, j^* from gene *j* to gene *i*, i.e., the system gain from *x_j_*(*t*) to *x_i_*(*t*) γ*^i, j^*_0_ = $\sup\;\frac{{\left\|x_{i}(t)\right\|}_{2}}{{\left\|x_{j}(t)\right\|}_{2}}$, can be estimated by solving the following LMI-constrained optimization problem:

(30) $$\begin{array}{c} \gamma_{o}^{i,j}=\begin{array}{cc} \underset{P>0}{\min} & \rho \end{array}\\ \text{subject to }\left[\begin{array}{cc} A_{j}^{T}PA_{j}-P+D_{i}^{T}D_{i} & A_{j}^{T}PB_{j}\\ B_{j}^{T}PA_{j} & B_{j}^{T}PB_{j}-\rho I \end{array}\right]\leq 0 \end{array}$$

Based on the above analysis, we can easily calculate the regulatory information flow in Equation (29) and solve the LMI-constrained optimization in Equation (30) for the information transductivity γ_0_*^i, j^* between any two genes in a GRN if we identify the system parameter *A* in Equation (26) from the microarray temporal data.

### Estimation of information transductivity of a GRN by microarray sample data

If the microarray data for measuring the information flow of the GRN in Figure 2 are of one time-point microarray from different samples, then the regression model for gene regulation in Equation (24) is modified to the following:

(31) $$x_{i}(k)=a_{i,1}x_{1}(k)+\cdots +a_{i,n}x_{n}(k)+v_{i}(k),for\;k=1,\ldots ,K$$

where *x*_1_(*k*),…, *x_n_*(*k*) denote the gene expression levels of the GRN in Figure 2 at the *k*th sample microarray. Therefore, the whole GRN in Figure 2 can be represented by:

(32) X(k)=AX(k)+v(k), for k=1,…,K

where $\begin{array}{l} X(k)\;=\;\left[\begin{array}{c} x_{1}(k)\\ \vdots \\ x_{n}(k) \end{array}\right],\;v(k)\;=\;\left[\begin{array}{c} v_{1}(k)\\ \vdots \\ v_{n}(k) \end{array}\right] \end{array}$, and $\begin{array}{l} \;A\;=\;\left[\begin{array}{ccc} a_{1,\;1} & \cdots & a_{1,\;n}\\ \vdots & \ddots & \vdots \\ a_{n,\;1} & \cdots & a_{n,\;n} \end{array}\right] \end{array}$.

Suppose we want to calculate the information flow from gene *j* to gene *i* of the GRN in Figure 2. Then, Equation (32) needs to be re-arranged as

(33) $$\begin{array}{l} X(k)=AjX(k)+Bjxj(k)\\ \;xi(k)=DiX(k) \end{array}$$

where *A_j_, B_j_*, and *D_j_* are defined in Equations (27) and (28).

Then, from Equation (33), we can get the information flow from gene *j, x_j_*(*k*), to gene *i, x_i_*(*k*), as follows:

(34) $$x_{i}\left(k\right)\;=D_{i}{\left(I-A_{j}\right)}^{-1}B_{j}x_{j}\left(k\right)$$

Hence, the regulatory information transduction equation from gene *j* to gene *i* is given by:

(35) $$\frac{x_{i}(k)}{x_{j}(k)}=T_{i,j}=D_{i}{\left(I-A_{j}\right)}^{-1}B_{j}$$

Then the information transductivity from gene *j* to gene *i* is given by

(36) $$\gamma_{0}^{i,j}=|D_{i}{\left(I-A_{j}\right)}^{-1}B_{j}|$$

Therefore, if we can identify regulatory parameter *a*_*i*, 1_,…, *a_i, n_* in Equation (31) by the recursive least square estimation algorithm in Equation (20) through microarray data with *K* samples for all genes of a GRN, we can identify the regulatory matrix *A* in Equation (32) and then *A_j_, B_j_*, and *D_i_*. In this situation, we can estimate the information transduction equation *T_i, j_* in (35) from gene *j* to gene *i* and information transductivity γ_0_*^i, j^* in Equation (36) for any *i* and *j*.

**Remark 5**: (i) MicroRNA-mediated repressions can be also involved in our model if NGS data is available. The repressions are considered as the extra inhibition term −*B_m_m*(*t*) in Equation (26). *m*(*t*) denotes the expressions of microRNAs. In this case, Equation (26) is modified as:

$$X\text{(}t+\text{1) = }AX\text{(}t\text{)}-B_{m}m\text{(}t\text{)}+v\text{(}t\text{)}$$

Then, Equation (28) is modified as:

$$\{\begin{array}{ccc} X(t+1) & = & A_{j}X(t)+B_{j}x_{j}(t)-B_{m}m(t)+v(t)\\ \;x_{i}(t) & = & D_{i}X(t)\; \end{array}$$

Therefore, we obtain

$$x_{i}(t)=\sum_{k=0}^{t}D_{i}A_{j}^{t-k}\left[B_{j}x_{j}(k)-B_{m}m(k)+v(k)\right]$$

In this case, the information transductivity γ_0_*^i, j^* = $\sup\;\frac{{\left\|x_{i}(t)\right\|}_{2}}{{\left\|x_{j}(t)\right\|}_{2}}$ from gene *j* to gene *i* is the same as found in Equation (30). However, the gene expression *x_i_*(*t*) with microRNA-mediated repressions is different from that without the repressions due to the extra inhibition term −*B_m_m*(*t*) in the above equation. (ii) For the system identification of Equation (13), (19), (26), or (32), the noise term, *w_i_* or *v*, is also model residue. In other words, it represents the modeling error and environmental noise. In general, it cannot be measured beforehand, and is corrupted in microarray data. After the system parameter *θ* was estimated by the system identification in Equation (14), the noise could be estimated by the modeling error $\hat{w}$ = *y* −ϕ $\hat{\theta }$, where $\hat{\theta }$ is the estimate of *θ*, from Equation (13) and Equation (19).

## Example of calculating information flow in signal transduction pathways and control of a GRN

**Figure 3:**
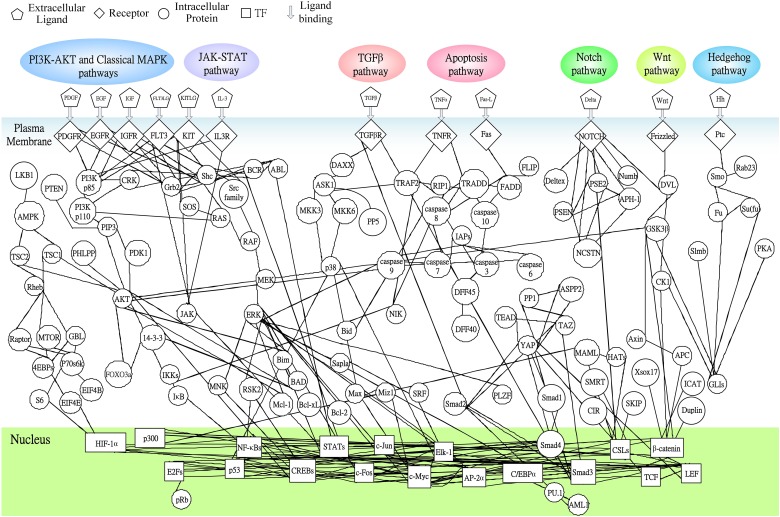
**The coupling signal transduction pathways that control gene activities related to leukemogenesis**. The figure consists of 147 groups of proteins and 18 groups of TFs. The downstream network in the nucleus denotes the gene regulatory network as shown in Figure 4, while the pathways in plasma denote protein-protein interactions. Other notations are defined and shown at the top of this figure.

**Figure 4:**
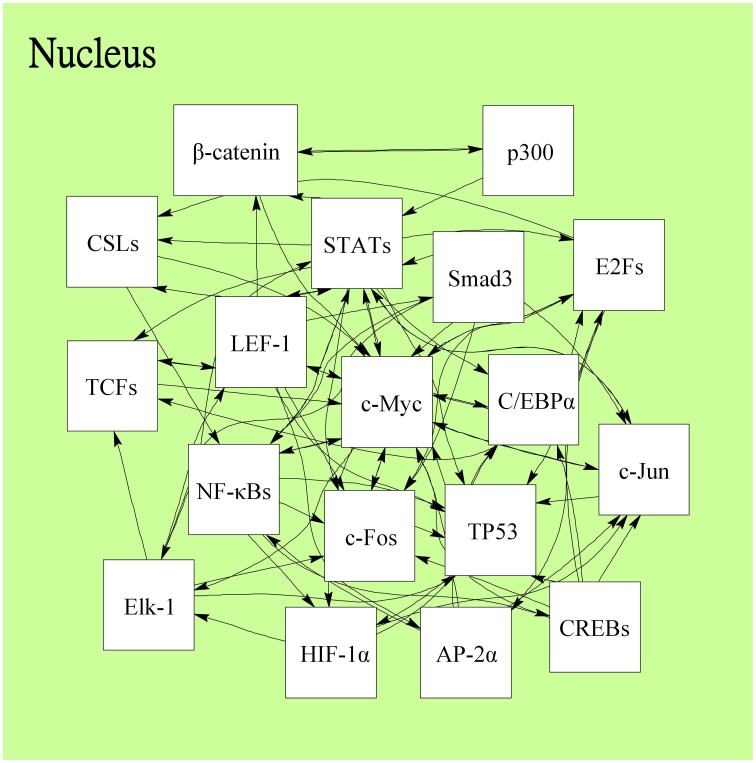
**The information flow in the gene regulatory network related to leukemogenesis in the downstream of coupling signal transduction pathways in Figure 3**. Each TF regulates other genes and acts as a gene of TF regulated by other TFs. The 18 leukemogenesis-related TFs and the regulations are determined by the TRANSFAC gene-regulation database and shown as follows: AP-2α, β-catenin, C/EBPα, p300, c-Myc, CREBs (ATF-4/CREB1/Luman/OASIS/CREB3L2/CREB-H/ AIBZIP/CREBPA), CSLs(RBP-JK/RBPJL), E2Fs (E2F1/E2F2/E2F3), Elk-1, c-Fos, HIF-1α, c-Jun, LEF-1, NF-κBs (NF-κB1/NF-κB1-p50/NF-κB2-p52, NF-κB2/ NF-κB2-p52/c-Rel/RelA-p65/RelB), Smad3, STATs (STAT1/STAT3/STAT5), TCFs (TCF-1/TCF-3/TCF-4), and p53.

**Figure 5:**
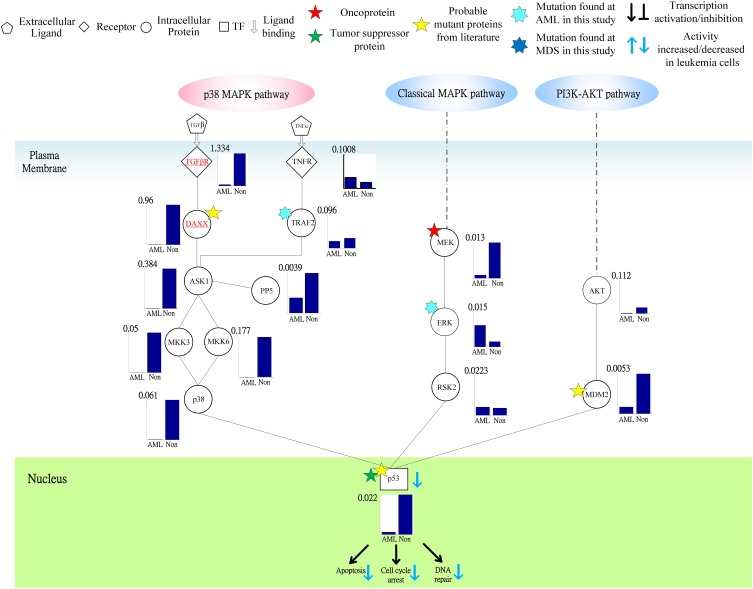
**Signal transductivities of the proteins in p38 MAPK and PI3K-AKT coupling pathways contributing to loss of transductivity of TF p53 at the acute myeloid leukemia (AML) subtype when compared with normal type**. The solid line represents the PPIs in the plasma membrane, while the dot-and-dash line represents the interactive contribution from the other pathways. The proteins underlined in red play an important role in dysfunction of the downstream TF. The other notations are shown at the top of the Figure. Stars ★ denote the locations at which the genetic mutations occur.

**Figure 6:**
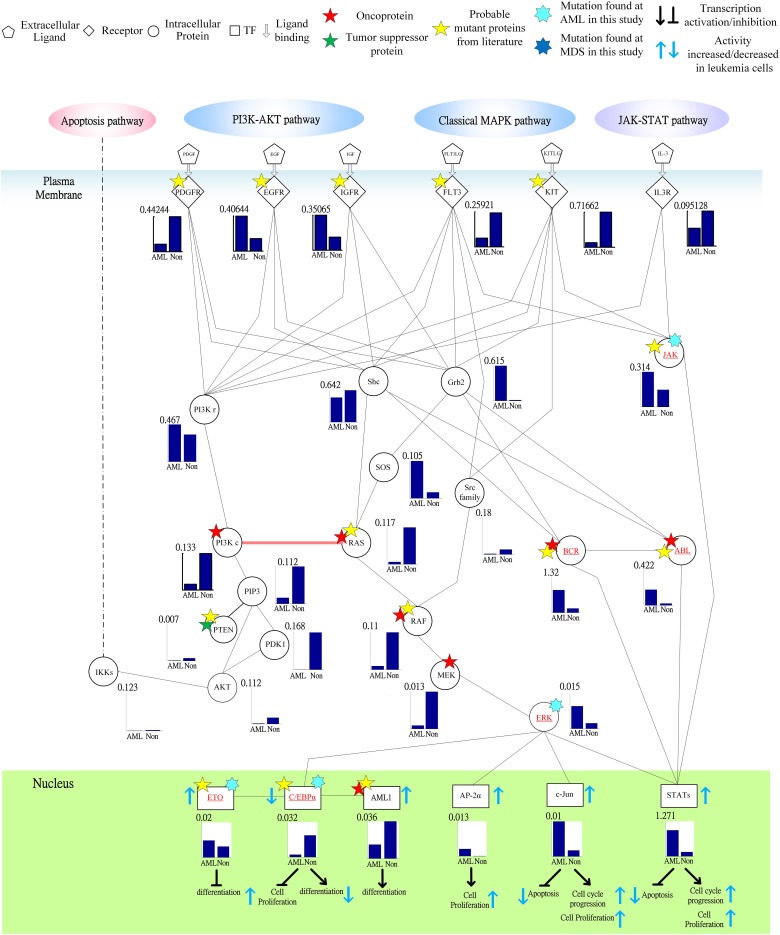
**Signal transductivity of the proteins in JAK-STAT and MAPK coupling pathways contributing to the gain of transductivity of STATs and the loss of transductivity of CEBPα at the acute myeloid leukemia (AML) subtype**. The notations are the same as those in Figure 5. The bold solid line with a pink arrow denotes the demonstration of the directional signal flow between two pathways.

Following on from the analyses of signal transductivity of coupling signal transduction pathways based on protein–protein interaction data from the Kyoto Encyclopedia of Genes and Genomes (KEGG) pathway database (Kanehisa and Goto, 2000; Kanehisa et al., 2014) and the information transductivity of a GRN based on the GRN from the TRANSFAC gene-regulation database (Matys et al., 2006) in the above section, we applied the microarray data for estimation of the signal transductivities of the signal transduction pathways in normal cells and cancer cells (Figure 3), and compared the differences between these to determine the dysfunction in information flow due to carcinogenesis. Furthermore, we estimated the information transductivities between different genes in a GRN in normal and cancer cells (Figure 4). The proteins with dysfunctional information flow in signal transduction pathways and a GRN can be considered as therapeutic targets. Using the microarray sample raw data from the Gene Expression Omnibus (GEO) database (accession number: GSE 13159) (Haferlach et al., 2010) as *y*(*k*) and the data of the corresponding ligand for each receptor protein as *u*(*k*) in Equation (15), we used the recursive least square parameter estimation method to identify the system parameters *C, H*, and *B* in Equation (15) of the coupling transduction pathways shown in Figure 3. The estimated signal transductivities from extracellular signals to 28 TFs in acute myeloid leukemia (AML) cancer cells and non-leukemia normal cells are given in Table 1. Among them, the signal transductivity of each protein in the MAPK and PI3K-SKT coupling pathways in normal and cancer cells is shown in Figure 5. The dysfunction in signal transductivity in a protein is mainly due to genetic mutations involved in the carcinogenesis. The effects of these genetic changes on cellular function, via signal transductivity changes in TFs, are also shown in Figure 5. Similarly, the signal transductivity of each protein in the MAPK and JAK-STAT coupling pathways in normal and cancer cells is shown in Figure 6. The effects of signal transductivity changes in TFs on cellular functions are also shown in Figure 6. The information transductivity of the GRN related to leukemia is shown in Figure 4, based on the GRN from the TRANSFAC gene-regulation database (Matys et al., 2006). By using microarray sample raw data (accession number: GSE 13159) (Haferlach et al., 2010) as *X*(*k*) to identify the system matrix *A* of the GRN in Equation (32), we estimated the information flow γ_0_*^i, j^* in Equation (36) between any two genes *i* and *j* in the GRN from Equation (36). For example, the information flow γ_0_*^i, j^* from *STATs* to *c-Jun* is 0.3104 in AML cancer cells and 3.3952 in normal cells; the information flow γ_0_*^i, j^* in Equation (36) from *CREBs* to *p53* is 0.6276 in AML cancer cells and 0.2273 in normal cells. This clearly shows that the information flow in a GRN can be affected by leukemia. Additionally, according to the results of parameter estimation methods in AML and non-leukemia cells, the correlation coefficients of noise *v*(*k*) and the identified parameter *A* in Equation (32) in AML cells almost lie within −0.2 and +0.2 (96.71%,), while those in non-leukemia cells almost lie within −0.2 and +0.2 (98.58%), i.e., the estimation parameters are almost uncorrelated with noises.

**Table 1:** **The signal transductivities of signal transduction pathways in Figure 2 from extracellular signals to 28 TFs in non-leukemia normal cells and acute myeloid leukemia (AML) cancer cells**.

**TFs**	**Signal transductivity**	
	**Non-leukemia**	**AML**
CSLs	0.0059	0.2267
P300	0.0002	0.0065
CTBPs	0.2330	0.1233
AP-2α	0.0001	0.0027
STATs	0.1551	0.9443
c-Jun	0.0017	0.0096
β-catenin	0.0160	0.0659
LEF-1	0.2295	0.0537
TCFs	0.0198	0.0096
c-Myc	0.0257	0.0016
C-Fos	0.0003	0.0000
CREBs	0.0168	0.0029
Elk-1	0.0321	0.0057
P53	0.0219	0.0013
FOXO3a	0.0050	0.0000
NF-κBs	0.0052	0.0001
HIF-1α	0.0000	0.0000
E2Fs	0.0001	0.0000
pRb	0.0003	0.0001
C/EBPα	0.0196	0.0023
AML1	0.0357	0.0134
ETO	0.0064	0.0099
PLZF	0.0011	0.0020
PU.1	0.0041	0.0072
Smad2	0.0373	0.0297
Smad3	0.4713	0.1089
Smad4	0.0150	0.1090
GLIs(Ci)	0.0803	0.0132

**Figure 7:**
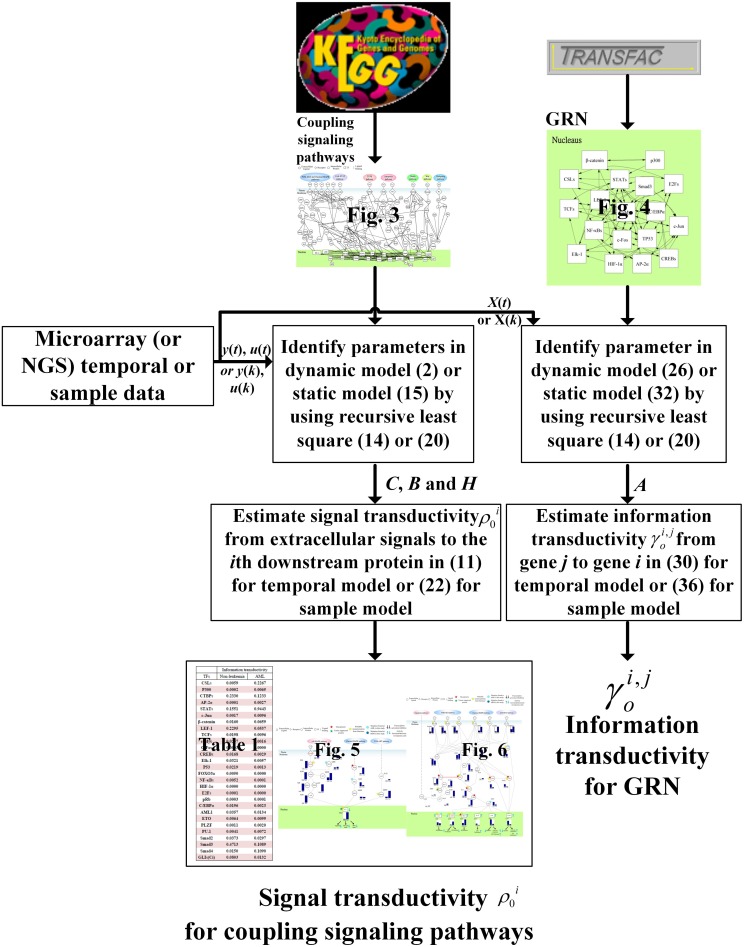
**The estimation flowchart of the signal transductivity for coupling signaling pathways and information transductivity for a GRN**. The figure summarizes parameter estimations and transductivity measurements of information flows in this study.

In order to clarify the proposed method for a broad audience, we use a flowchart in Figure 7 to simplify the estimation procedures of signal transductivity of coupling signaling pathways and information transductivity in a GRN. We also clarified the estimation procedure of the method proposed in this study. If we want to measure signal transductivity of coupling signal transduction pathways and information transductivity in a GRN, some technical specifications are required. First, we need microarray sample data (or temporal data) or NGS sample data (or temporal data) for normal or cancer cells. Second, we need recursive parameter estimation algorithm in Equation (12) or (20), and singular value decomposition and basic matrix operation for Equations (18), (23), and (36). Finally, LMITOOL is required for solving the LMI-constrained optimization problem in Equation (30) (Elghaoui et al., 1995).

In order to estimate the signal transductivities of the coupling signal transduction pathways (Figure 3) obtained from the KEGG database and the information transductivities of the GRN (Figure 4) obtained from the TRANSFAC database in non-leukemia normal cells and AML cancer cells, we applied the microarray sample data from non-leukemia normal cells or AML cancer cells to the Equations (15) and (32). We first identified the system parameters in the models by using the recursive least square parameter estimation algorithm in Equation (20). Finally, we use Equations (22) and (36) to estimate the signal transductivities of coupling signaling pathways in Table 1 and Figures 5, 6 and information transductivities in the GRN, respectively.

## Conclusion

In this study, the signal transductivity and information transductivity in cellular networks were estimated using microarray temporal data and a state-space model of signal processing systems. If the microarray data are obtained from different samples with a single time point, the static state-space model can also be developed to measure the information flow of cellular systems from multi-input extracellular signals to multi-output TFs in the coupling signal transduction pathways. Furthermore, we also proposed an input/output state-space signal model to overcome the difficulties of the digraph theory method in efficiently estimating the regulatory one-gene-to-another in a complex digraph network of a GRN. Finally, the proposed signal transductivity and information transductivity methods were applied to measure the signal transductivity of coupling signal transduction pathways and the information transductivity of a GRN related to cancer via microarray sample data. By comparing signal transductivity and information transductivity between cancer and normal cells, we were able to determine the systematic dysfunctions of proteins and genes in the signal transduction pathways or a GRN easily, using the proposed method and microarray data. Since the proposed information flow models can also be identified using high-throughput data such as NGS, real-time PCR, or proteomic data, the proposed model has great potential for efficiently estimating the signal transductivity and information transductivity of cellular systems using such data. However, we do not discuss the use of differential expression protein data here.

### Conflict of interest statement

The authors declare that the research was conducted in the absence of any commercial or financial relationships that could be construed as a potential conflict of interest.

## Appendix

### Appendix A

Let us denote the Lyapunov function of the coupling signal transduction pathways in Equation (4) as *V*($\tilde{y}$(*t*)) = $\tilde{y}$*^T^*(*t*)*P*$\tilde{y}$(*t*), for some symmetric positive definite matrix *P* = *P^T^* > 0. We have:

(A1) $$\begin{array}{l} V(\tilde{y}(t+1))={\tilde{y}}^{T}(t+1)P\tilde{y}(t+1)={\left\{C\tilde{y}(t)+Bu(t)\right\}}^{T}\\ P\left\{C\tilde{y}(t)+Bu(t)\right\}={\tilde{y}}^{T}(t)C^{T}PC\tilde{y}(t)+u^{T}(t)B^{T}PC\tilde{y}(t)+\\ {\tilde{y}}^{T}(t)C^{T}PBu(t)+u^{T}(t)B^{T}PBu(t)-\rho u^{T}(t)u(t)-{\tilde{y}}^{T}\\ \left(t)P\tilde{y}(t)+{\tilde{y}}^{T}(t)\tilde{y}(t)-{\tilde{y}}^{T}(t)\tilde{y}(t)+{\tilde{y}}^{T}(t)P\tilde{y}(t)+\rho u^{T}(t)u(t\right) \end{array}$$

If we assume

(A2) $$\begin{array}{l} {\tilde{y}}^{T}(t)C^{T}PC\tilde{y}(t)+u^{T}(t)B^{T}PC\tilde{y}(t)+{\tilde{y}}^{T}(t)C^{T}PBu(t)\\ +u^{T}(t)B^{T}PBu(t)-\rho u^{T}(t)u(t)-{\tilde{y}}^{T}(t)P\tilde{y}(t)+\\ {\tilde{y}}^{T}(t)\tilde{y}(t)-{\tilde{y}}^{T}(t)\tilde{y}(t)\leq 0 \end{array}$$

Then, Equation (A1) becomes

(A3) $${\tilde{y}}^{T}(t+1)P\tilde{y}(t+1)\leq {\tilde{y}}^{T}(t)P\tilde{y}(t)+\rho u^{T}(t)u(t)$$

Summing the above inequality from *t* = 0 to *t_p_*, we get

(A4) $$\begin{array}{l} {\tilde{y}}^{T}(t+1)P\tilde{y}(t+1)\leq {\tilde{y}}^{T}(0)P\tilde{y}(0)-\sum_{t=0}^{t_{p}}{\tilde{y}}^{T}(t)\tilde{y}(t)+\\ \;\rho \sum_{t=0}^{t_{p}}u^{T}(t)u(t) \end{array}$$

By the factor $\tilde{y}$(0) = 0, we get

(A5) $$\sum_{t=0}^{t_{p}}{\tilde{y}}^{T}(t)\tilde{y}(t)\leq \rho \sum_{t=0}^{t_{p}}u^{T}(t)u(t)$$

which in Equation (6).

The above inequality holds if the assumption of the inequality in Equation (A2) holds, i.e.,

(A6) $$\left[\begin{array}{cc} {\tilde{y}}^{T}(t) & u^{T}(t) \end{array}\right]\left[\begin{array}{cc} C^{T}PC-P+I & C^{T}PB\\ B^{T}PC & B^{T}PB-\rho I \end{array}\right]\left[\begin{array}{c} \tilde{y}(t)\\ u(t) \end{array}\right]\leq 0$$

i.e., if the LMI in Equation (7) holds, then the coupling signal transduction pathways in Equation (4) have a transduction level (Equation 6).
